# Combined effects of elevated temperature and *Deepwater Horizon* oil exposure on the cardiac performance of larval mahi-mahi, *Coryphaena hippurus*

**DOI:** 10.1371/journal.pone.0203949

**Published:** 2018-10-17

**Authors:** Prescilla Perrichon, Edward M. Mager, Christina Pasparakis, John D. Stieglitz, Daniel D. Benetti, Martin Grosell, Warren W. Burggren

**Affiliations:** 1 Department of Biological Sciences, University of North Texas, Denton, Texas, United States of America; 2 Department of Marine Biology and Ecology, University of Miami, Rosenstiel School of Marine and Atmospheric Science, Miami, Florida, United States of America; 3 Department of Marine Ecosystems and Society, University of Miami, Rosenstiel School of Marine and Atmospheric Science, Miami, Florida, United States of America; University of Siena, ITALY

## Abstract

The 2010 *Deepwater Horizon* oil spill coincided with the spawning season of many pelagic fish species in the Gulf of Mexico. Yet, few studies have investigated physiological responses of larval fish to interactions between anthropogenic crude oil exposure and natural factors (e.g. temperature, oxygen levels). Consequently, mahi mahi (*Coryphaena hippurus*) embryos were exposed for 24 hours to combinations of two temperatures (26 and 30°C) and six concentrations of oiled fractions of weathered oil (from 0 to 44.1 μg ∑50PAHs·L^-1^). In 56 hours post-fertilization larvae, heart rate, stroke volume and cardiac output were measured as indicators of functional cardiac phenotypes. Fluid accumulation and incidence of edema and hematomas were quantified as indicators of morphological impairments. At both 26 and 30°C, oil-exposed larvae suffered dose-dependent morphological impairments and functional heart failure. Elevation of temperature to 30°C appeared to induce greater physiological responses (bradycardia) at PAH concentrations in the range of 3.0–14.9 μg·L^-1^. Conversely, elevated temperature in oil-exposed larvae reduced edema severity and hematoma incidence. However, the apparent protective role of warmer temperature does not appear to protect against enhanced mortality. Collectively, our findings show that elevated temperature may slightly decrease larval resilience to concurrent oil exposure.

## Introduction

On April 20^th^ 2010, the *Deepwater Horizon* (*DWH*) offshore drilling platform exploded and resulted in the loss of crude oil, methane and other gases from 1500 m below the sea’s surface into the northern Gulf of Mexico. The oil from *DWH* was a light crude oil containing saturated n-alkanes, polycyclic aromatic hydrocarbons (PAHs) and their alkylated homologues, with over 50% comprising low-molecular-weight hydrocarbons (methane and C2-C11) [1]. Immediately after discharge, natural weathering processes generated changes in the physico-chemical properties of source oil (considered “light oil”) in the seawater column [1,2]. These alterations in composition are generally due to processes such as evaporation of volatile compounds (e.g. high water temperature), emulsification, natural dispersion by oceanic dynamics (e.g. winds, currents), dissolution of soluble compounds, photooxidation by solar irradiance (UV), sedimentation, interaction with fine particles and biodegradation by environmental microorganisms [3,4]. Consequently, the vertical transport of oil from the site of release to the sea surface and the associated weathering processes generated weathered oil that was significantly more toxic than oil released at the well head, with proportionally higher molecular weight PAHs [5,6].

The timing of the *DWH* incident coincided with the spawning and larval development of various commercial and recreational species such as mahi-mahi (*Coryphaena hippurus*) (hereafter referred as “mahi”) and so likely resulted in oil exposure to early life stages [7–9]. Both crude and weathered oil-derived PAHs adversely affect morphology and development of fish embryos and larvae at low μg.L^-1^ aqueous concentrations [5,10–22]. For decades, clear evidence has indicated that the cardiovascular system of fishes at the onset of organogenesis is a major primary target of PAHs and particularly of the tricyclic families of PAHs [11,18,21,23–28]. Through different mechanisms such as blockade of potassium currents or disruption of intracellular calcium cycling during excitation-contraction coupling [29], these PAHs cause a suite of pathologies related to embryonic heart failure, mostly including bradycardias and arrhythmias [18,19,25,30,31]. These functional cardiac impairments are often accompanied with high incidence of edemas and improper cardiac chamber looping, shape or orientation [18,20,32]. At high doses, these impairments may induce increased injuries, developmental delay or mortality [11,25,30].

The physiological mechanism of cardiac impairment in individual larval fish are still not well understood. Most studies have focused on the single measurement of heart rates. Heart rate is relatively simply and accurately measured, but is not a holistic indicator of cardiac performance. For example, in young adult mahi exposed to *DWH* oil, heart rate measured *in situ* was unaffected by oil exposure, but stroke volume, cardiac output and heart stroke work were all significantly diminished [33]. Cardiac output also decreases in response to oil exposure in larval stages of the coastal red drum (*Sciaenops ocellatus)* [14]. Understanding how not just heart rate, but also stroke volume and the all-important cardiac output, are influenced by oil exposure will add physiological understanding as to whether and how fishes can maintain homeostasis and enhance chances for survival.

The cardiovascular system of larval fishes will likely respond to a multitude of environmental stressors [34]. Some of these natural stressors may act synergistically with environmental toxicants. Thus, for example, elevated temperatures will increase metabolism, which may then increase the susceptibility of organisms to oil exposure. However, few studies have explored the synergistic/antagonistic relationships between multiple natural and anthropogenic stressors [35–37].

Mahi inhabit waters of the northern Gulf of Mexico [9,38,39]. Hence, their newly-fertilized, buoyant eggs could have potentially been exposed to crude oil from *Deepwater Horizon*. Moreover, during that incident, temperatures of surface waters ranged from 25 to 30°C [40]. Here, we investigated how weathered *DWH* oil exposure interacts with elevated temperatures to influence the cardiac performance of larval mahi. We hypothesized that mahi would be more susceptible to weathered oil toxicity when is co-exposed to high rearing temperature. Elevated temperature may exacerbate functional cardiac disruption especially as upper physiological thermal limits are approached. To test this hypothesis, embryonic mahi were co-exposed to different sublethal concentrations of high energy water accommodated fractions (HEWAFs) of oil in combination with two rearing temperatures (26 and 30°C). Morphological edemas and cardiac physiological variables were then monitored in the larval stages.

## Materials and methods

### Preparation of oil exposure solutions

All oil samples were collected under chain of custody during the *DWH* event and kept at the University of Miami. Oil from the surface (OFS) collected on July 29, 2010 via skimming operations was used to prepare the high energy accommodated fraction (HEWAF). HEWAF was prepared at a loading rate of 1 g of oil per liter of UV-sterilized seawater (35 ppt) and mixed in a Waring CB15 blender (Torrington, CT) at low speed for 30 s. The HEWAF was generated at room temperature and was not filtered prior to use. The mixture was immediately transferred to a glass separatory funnel and left to settle for 1 h. The 90% lower portion of the solution was carefully drained and retained for subsequent use as 100% HEWAF. This fraction was then diluted for test exposures. Dilution solutions were mixed on a stir plate (180 rpm) for 5 min and then aliquoted for test exposures. The exposure tests consisted of five sublethal concentrations (0.5, 1, 2, 4 and 8% HEWAF), and included a seawater control.

### Maintenance and egg production of mahi

Mahi broodstock were captured in the offshore waters of the Straits of Florida off the coast of Miami (FL, USA) in the general coordinates of 25° 34.000’N / 80° 00.000’W using hook and line angling (special Activity License #: SAL-15-0932B-ABC). Broodstock age and growth metrics are detailed in Stieglitz et al. (2017). The fish were subsequently transferred to the University of Miami Experimental Hatchery (UMEH), where they were acclimated in 80 m^3^ fiberglass maturation tanks equipped with partially recirculated and temperature controlled water at 26–27°C [41]. All embryos used in the experiments described herein were collected within 2–10 h following a volitional (non-induced) spawn using standard UMEH methods [42]. The embryos used in the experiments came from a total of six different volitional spawning events over time. These spawning events involved three different groups of wild mahi, with each group consisting of unrelated (i.e. non-sibling) adult wild mahi. Sex ratios during the spawning events ranged from 1–5 females to one male, depending on the spawning group of fish. Over the course of the experiments there were three males and eight females involved in the spawning events producing embryos. In each replicated experiment, embryos from the same batch were exposed to both low (26°C) and high temperature (30°C) treatments to avoid any confounding effects that could potentially be related to batch or family variability. Additionally, the experiments were conducted using a multitude of spawns (six total) from three different spawning groups (“families”, though unrelated) of broodstock fish, thereby minimizing any potential occurrence of confounding effects due to family.

Following collection of eggs, a prophylactic formalin treatment (37% formaldehyde solution at 100 μl·L^-1^ for 1 h) was administered to the embryos, followed by 30 min of flushing with a minimum of 300% water volume in the treatment vessel using filtered, UV-sterilized seawater. A small sample of eggs was collected from each spawn to microscopically assess fertilization rate and embryo quality. Spawns demonstrating low fertilization rate (< 85% hatching) or more than 5% developmental abnormalities were rejected.

### Ethics statements

Fishing for the mahi-mahi broodstock was done in accordance with the Florida Fish and Wildlife Conservation Commission—Special Activity License #: SAL-15-0932B-ABC which provides authorization for this activity. Fish capture, transport, and holding techniques were completed in accordance with the University of Miami Institutional Animal Care and Use Committee (IACUC) protocol numbers 15–019, 12–064, and 15–067.

### Embryonic exposure

**Fig 1:**
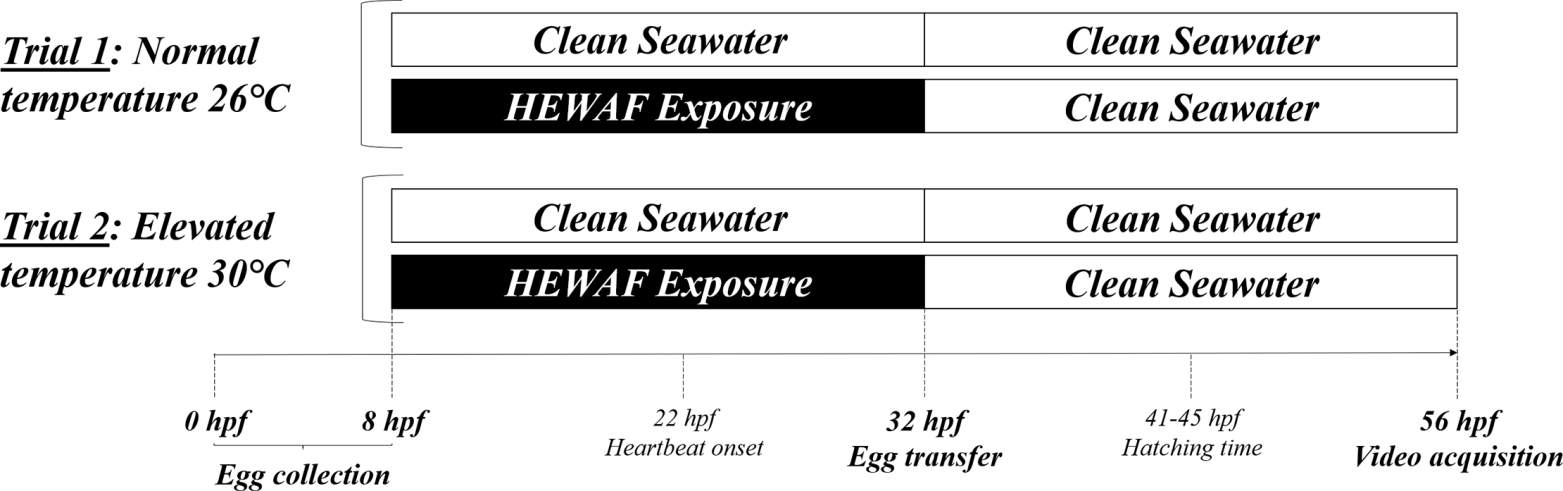
Experimental design. Mahi embryos (eight hpf) were exposed to HEWAF solutions during 24 hours and subsequently transferred to clean seawater for an additional 24 hours of development. Exposures were performed at two temperatures: a “normal” temperature of 26°C and an “elevated” temperature 30°C. Each temperature trial included a control group raised in seawater only. hpf; hours post-fertilization.

Dilutions of 100% HEWAF were distributed to 1 L-glass beakers for each concentration tested. 20 embryos of ~eight hours post-fertilization (hpf) per liter were randomly and equally distributed. Beakers were covered with a glass lid to prevent evaporation. Embryos were exposed to HEWAF fractions in triplicate for 24 h and then transferred to clean, oil-free seawater for a subsequent 24 hrs of development, during which time heart observations were made (Fig 1). Water quality was monitored daily, and included measurements of pH, dissolved oxygen, salinity and temperature (S1 Table). To assess the combined effects of oil exposure and temperature, trials were performed at two rearing temperatures (Fig 1) - 26 (”normal”) and 30°C (”elevated”)—in a temperature controlled environmental chamber with a 16:8 light/dark photoperiod. These temperatures represent the temperatures known to induce cardiovascular performance change in mahi embryos [43] and also represent the range of surface sea water temperatures typical of the Gulf of Mexico.

### Extraction and analyses of PAHs

Water samples were immediately collected in 250 mL amber glass bottles at 0 and 24 hours post-exposure. Samples were stored at 4°C and shipped overnight on ice to ALS Environmental (Kelso, WA, USA) for chemicals analysis. PAHs were then extracted and quantified by GC/MS-SIM (gas chromatography coupled to mass spectrometer-selective ion monitoring) following the US EPA method 8270D [44]. Blank analysis was carried out to ensure the absence of contamination prior and during analysis. Reported ∑PAH values represent the sum of 50 PAHs analytes selected for standard analysis for the Deepwater Horizon Natural Resource Damage Assessment toxicity testing program [45].

### Image and video capturing

In mahi at 26°C, the primitive heart (precursors) appears by 18–19 hpf, with the onset of heart beat appearing by 22 hpf. After hatching at 50–56 hpf, the sinus venosus and bulbus cordis start to differentiate. The heart starts to loop to the lateral side, as it begins to assume its adult configuration with formation of both valves around 80 hpf [43,46].

Cardiac function was assessed in larvae (56 hpf) by imaging techniques described by Perrichon et al. (2017a). Briefly, specimens were positioned on a thermal microscope stage temperature controller (Brook Industries, Lake Villa, IL). Unanaesthetized larvae were individually immobilized in a Petri dish containing 2% methylcellulose/98% seawater and orientated in a left lateral view. Images of specimens were captured using a Nikon SMZ800 stereomicroscope coupled to a Fire-i400 or Fire-i530c digital camera (Unibrain, San Ramon, CA). Images of perigastro-intestinal areas and 20 s long live videos were digitized at 30 frames.s^-1^ using PhotoBooth software. Calibrations were carried out using a stage micrometer. At the end of experiment, larvae were euthanized with an overdose of buffered tricaine methanesulfonate (MS-222).

### Morphological assessment

Measurements of edema and sinus venosus-yolk mass gap (SV-YM) were made using ImageJ software [47]. Edema area on the 2D images was measured as a proxy of the extent of pericardial edema present in larvae following the procedure described by Edmunds et al. (2015). The area was drawn with the ImageJ freehand tool enclosing the pericardial area plus the visible mass of yolk sac distortion (fluid accumulation). The SV-YM gap, which represents a measure of fluid accumulation, was determined by a line drawn from the posterior end of atrium to the yolk mass, after Edmunds et al. (2015). Incidence of internal hematomas was also scored. All abnormal blood accumulations out of the vascular route into the edema area were considered to be hematomas (S1 Fig).

### Quantification of ventricular cardiac function

Heart rate and stroke volume were determined from video sequences of the ventricle in 56 hpf larvae and used for cardiac output calculation—see Perrichon et al. (2017a) for methodology. Heart rate (heartbeat·min^-1^) was visually determined from slow speed videos.

Measurement of stroke volume in larval mahi was then determined by outlining the ventricular perimeter during end-diastole and end-systole [43,46]. All measurements were performed blind to treatment. Ventricular perimeter was fitted to the image with an ellipse using ImageJ software (Schneider et al., 2012; http://imagej.nih.gov/ij/, 1997–2016), where major and minor axes were then extracted. End-diastolic and end-systolic volumes of the ventricle were calculated using the conventional prolate spheroid formula:

$$Volume=\frac{4}{3}\pi \;{a\;b}^{2}$$

where *a* represents the major (longitudinal) semi-axis and *b* the minor (width) semi-axis [46].

For each larva, three successive systolic and diastolic cycles were captured, analyzed and then averaged to provide a representative estimator for ventricular volumes. The mean stroke volume (nL) was calculated as the difference between diastolic and systolic ventricular volumes. Cardiac output (nL·min^-1^) was calculated as the product of heart rate and stroke volume.

### Statistical analyses

Statistical analyses were performed using Statistica12 software. Data are expressed as mean ± standard error of mean (SEM). Data were standardized based on the average of four and three control groups at 26 and 30°C, respectively. Larvae were sampled per replicates and combined for measurement of ventricular function (S2 Table). Some videos with low quality were excluded from heart and stroke volume measurement. Results were statistically evaluated with multiple linear regressions (MLR) with temperature and concentrations as independent variables, as well their interaction on each morphometric and cardiac variable. When concentration effects were observed, ANOVAs were performed, followed by Tukey multiple comparison post hoc test. Pearson correlations were calculated to establish a potential link between the variation in cardiac variables and edema severity. A significance level of 5% was used for all analyses.

## Results

### PAH concentrations in HEWAF

Initial, final and equivalent geometric means of HEWAF concentrations are reported in Table 1 for both temperature trials. As predicted, ∑50PAHs concentrations linearly decreased with HEWAF dilutions in both temperature trials. PAH concentrations decreased by 9 to 30% at 26°C and by 15 to 32% at 30°C after 24 h exposure. However, the geometric means between concentrations at the normal 26°C and elevated 30°C temperatures were still quite similar.

**Table 1:** Initial and final HEWAF concentrations (μg.L^-1^) and their geometric means for both temperature trials.

		Concentrations (μg·L^-1^)			
Temperature	% HEWAF	Initial	Final	Geometric Mean	Loss
Normal 26°C	0	0.0	0.0	0.0	
	0.5	3.2	2.8	3.0	-11%
	1	8.0	6.7	7.4	-16%
	2	14.8	10.3	12.4	-30%
	4	32.6	29.7	31.2	-9%
	8	49.3	39.3	44.1	-20%
Elevated 30°C	0	0.0	0.0	0.0	
	0.5	3.4	2.8	3.1	-18%
	1	8.4	7.2	7.8	-15%
	2	18.0	12.3	14.9	-32%
	4	31.0	22.9	26.6	-26%
	8	57.2	40.3	48.8	-29%

Details of the chemical characterization of different HEWAF concentrations are indicated in S3 Table. All following physiological and morphological data are reported as geometric means of initial and final concentrations.

### Larval survival

Data presented in this manuscript are part of a complementary physiological study undertaken by Pasparakis et al. (2016) that focuses on sublethal effects of crude oil when combined with elevated temperature exposure. Consequently, no precise data on survival or hatching success are presented in the current manuscript. The combination of the highest HEWAF dilution (8% OFS) and the elevated rearing temperature of 30°C proved lethal for developing embryos [48]. Therefore, no data were acquired for biological analyses for this combination of exposures.

### Larval morphology

**Fig 2:**
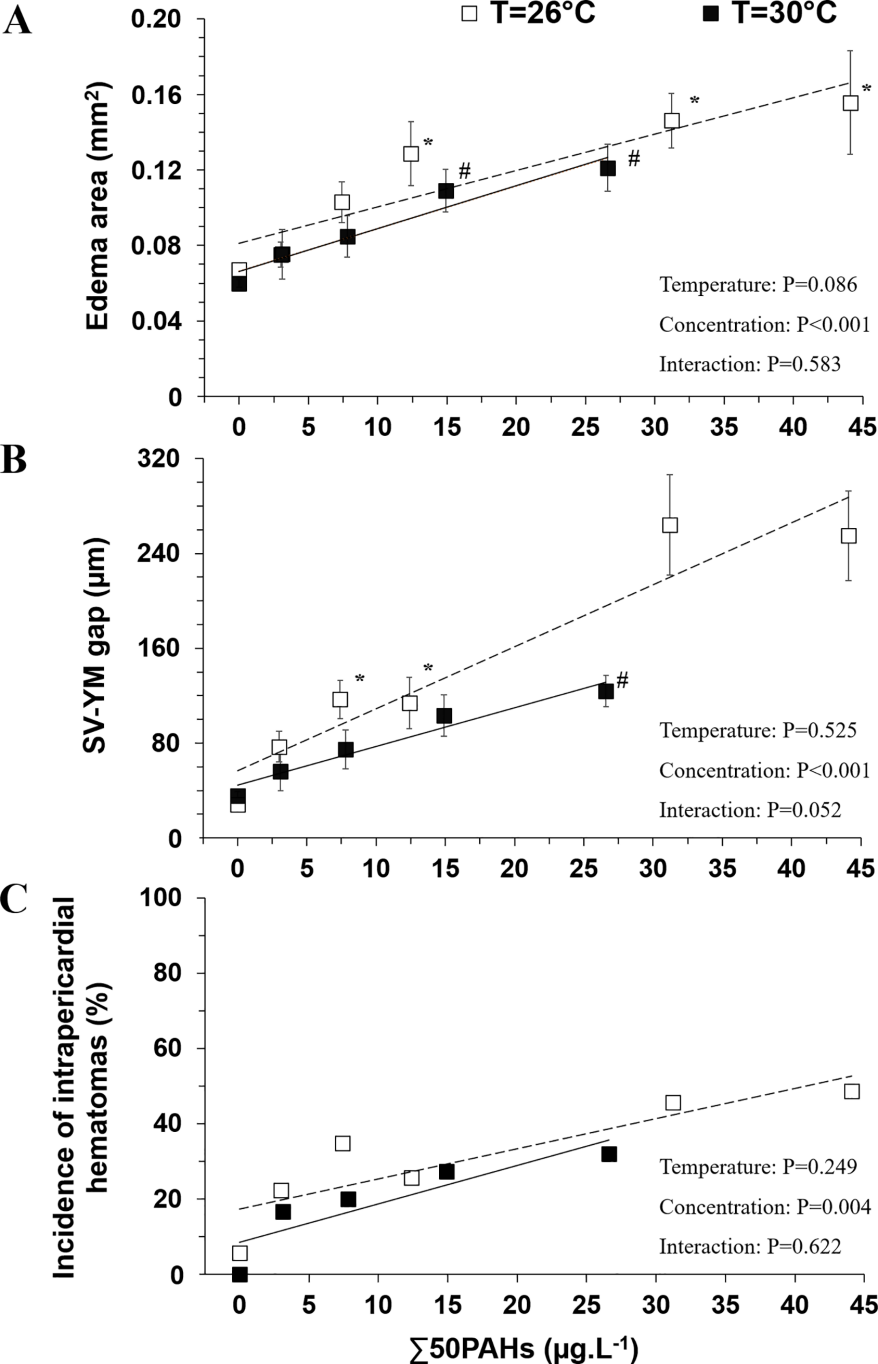
Morphological impairments associated with edema. (A) Edema area (mm^2^), (B) sinus venosus-yolk mass gap (μm) and (C) total incidence of intrapericardial hematomas measured at two rearing temperatures 26°C (N = 20–39) and 30°C (N = 20–56). Data for edema area and SV-YM gap are expressed as mean±SEM. Incidence of intrapericadial hematomas is expressed in total percent of hematomas scored in oil—exposed individuals. Simple linear regressions are given for graphical representation at both exposure temperatures. * (26°C) and # (30°C) indicate significant differences of oil exposure concentrations compared to respective control groups (P<0.05).

Larval mahi exposed to OFS HEWAF displayed a linear increase of edema area with increasing HEWAF concentrations (r^2^ = 0.30, P<0.001), irrespective of rearing temperature (Fig 2A). In both temperature conditions, edema area was significantly greater at PAH concentrations from 12.4 (26°C) and 14.9 (30°C) μg·L^-1^ compared to respective controls. However, multiple linear regression demonstrated no significant interaction (P = 0.583) between temperature and HEWAF concentration on the edema variable. The SV-YM gap in larvae raised at 26°C was ~4.0-fold greater than controls at 7.4 and 12.4 μg·L^-1^ and was more than 9-fold greater at 31.2 and 44.1 μg·L^-1^ (Fig 2B). Larvae raised at 30°C had a significantly greater SV-YM gap at the highest concentration compared to the control group. A significant main effect of PAH concentrations (P<0.001) and slight (but no significant) interaction (temperature x concentration) (P = 0.052) was observed on SV-YM gap, while no main effect of temperature (P = 0.525) was noted.

The occurrence of intrapericardial hematomas ranged linearly from 6% (control) to 49% (44.1 μg·L^-1^) in larvae raised at 26°C. At a rearing temperature of 30°C, the corresponding data were 0% (control) to 32% (26.6 μg·L^-1^) (Fig 2C). There was no significant interaction between temperature and oil concentration on hematomas incidence (P = 0.622).

### Ventricular function

**Fig 3:**
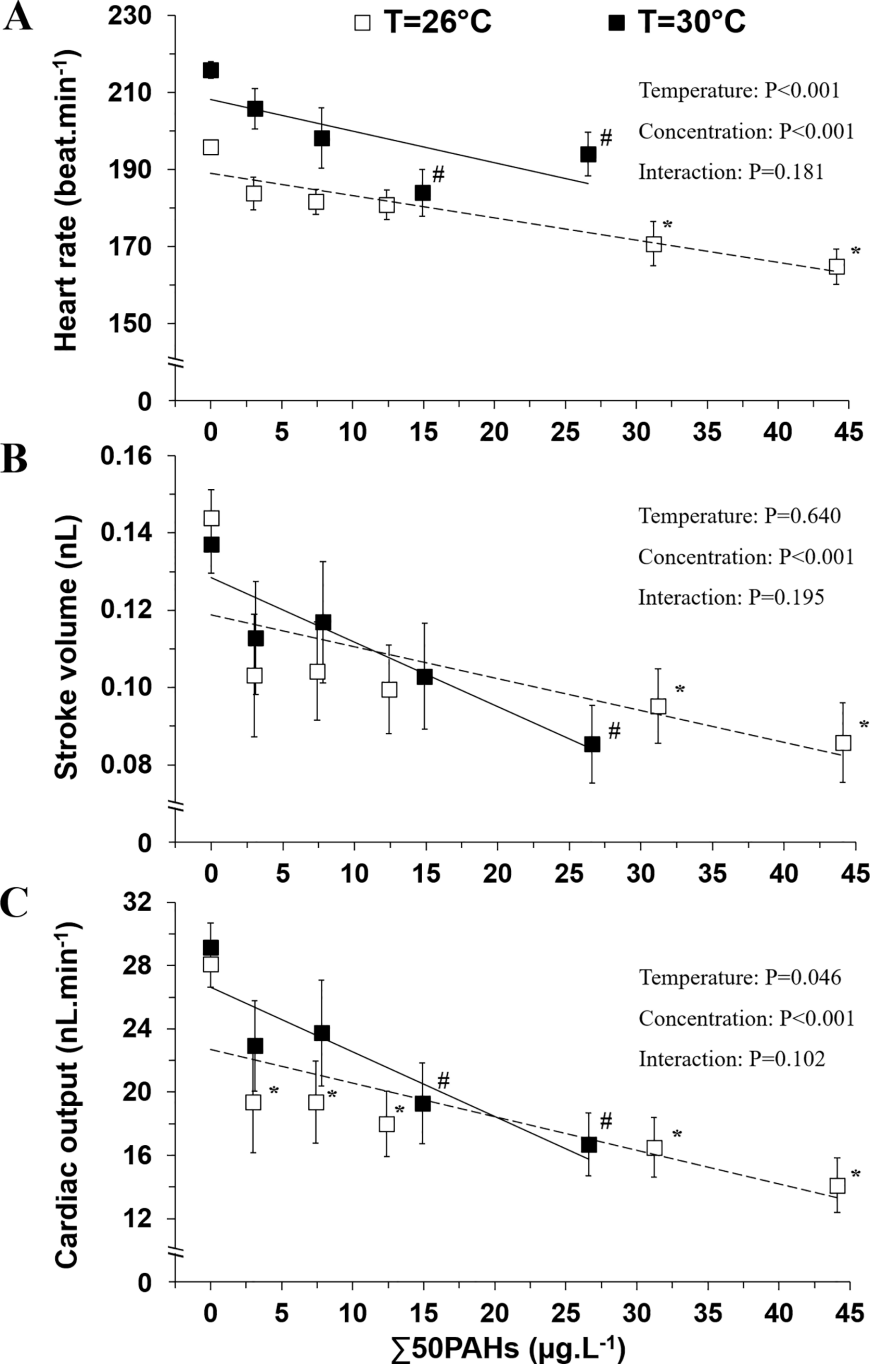
Cardiac variables measured in mahi larvae under combined oil and temperature exposures. (A) Heart rate (beat.min^-1^), (B) stroke volume (nL) and (C) cardiac output (nL·min^-1^) measured at two rearing temperatures 26°C (N = 20–39) and 30°C (N = 20–56). Data are presented as Mean±SEM. Simple linear regressions are given for graphical representation at both temperature exposures. * for 26°C and # for 30°C indicate significant differences of oil exposure concentrations compared to respective control groups (P<0.05).

Heart rate in control larvae was 10% higher at 30°C (216 beat·min^-1^) compared to 26°C (196 beat·min^-1^) (Fig 3A, pairwise comparison, P<0.001). At a rearing temperature of 26°C, heart rate was significantly decreased from control values by 13% at an exposure of 31.2 μg·L^-1^ and 16% at an exposure of 44.1 μg·L^-1^. At a rearing temperature of 30°C, heart rate was diminished by 10–15% over control values at an exposure of 14.9 μg·L^-1^. The MLR model had a predictive power of r^2^ = 0.30 with the temperature and concentration variables significantly contributing to the model (P<0.001). However, the interaction between temperature and HEWAF concentration was not significant (P = 0.181).

No change in stroke volume (pairwise comparison, P = 0.54) occurred in control larvae with rearing temperature (Fig 3B). Stroke volume was depressed in oil-exposed larvae at low concentrations for both temperature conditions. Specifically, stroke volume was significantly reduced by 34% from 31.2 μg·L^-1^ at 26°C and by 38% from 26.6 μg·L^-1^ at 30°C. However, temperature was not significant contributing factor of the MLR model (r^2^ = 0.08; temperature x concentration: P = 0.195).

Cardiac output also was not significantly different (pairwise comparison, P = 0.62) in both control groups raised at 26°C and 30°C (Fig 3C). However, there was a 31 to 50% decrease in cardiac output of oil-exposed larvae raised at 26°C. At 30°C, a significant decrease of 34% and 43% compared to controls was measured in larvae exposed to 14.9 and 26.6 μg·L^-1^, respectively. Temperature had slightly attenuated the cardiac output depression (P = 0.046), but the interaction (temperature x concentration) was not significant (P = 0.102).

### Correlation between functional and morphological oil-induced modifications

**Fig 4:**
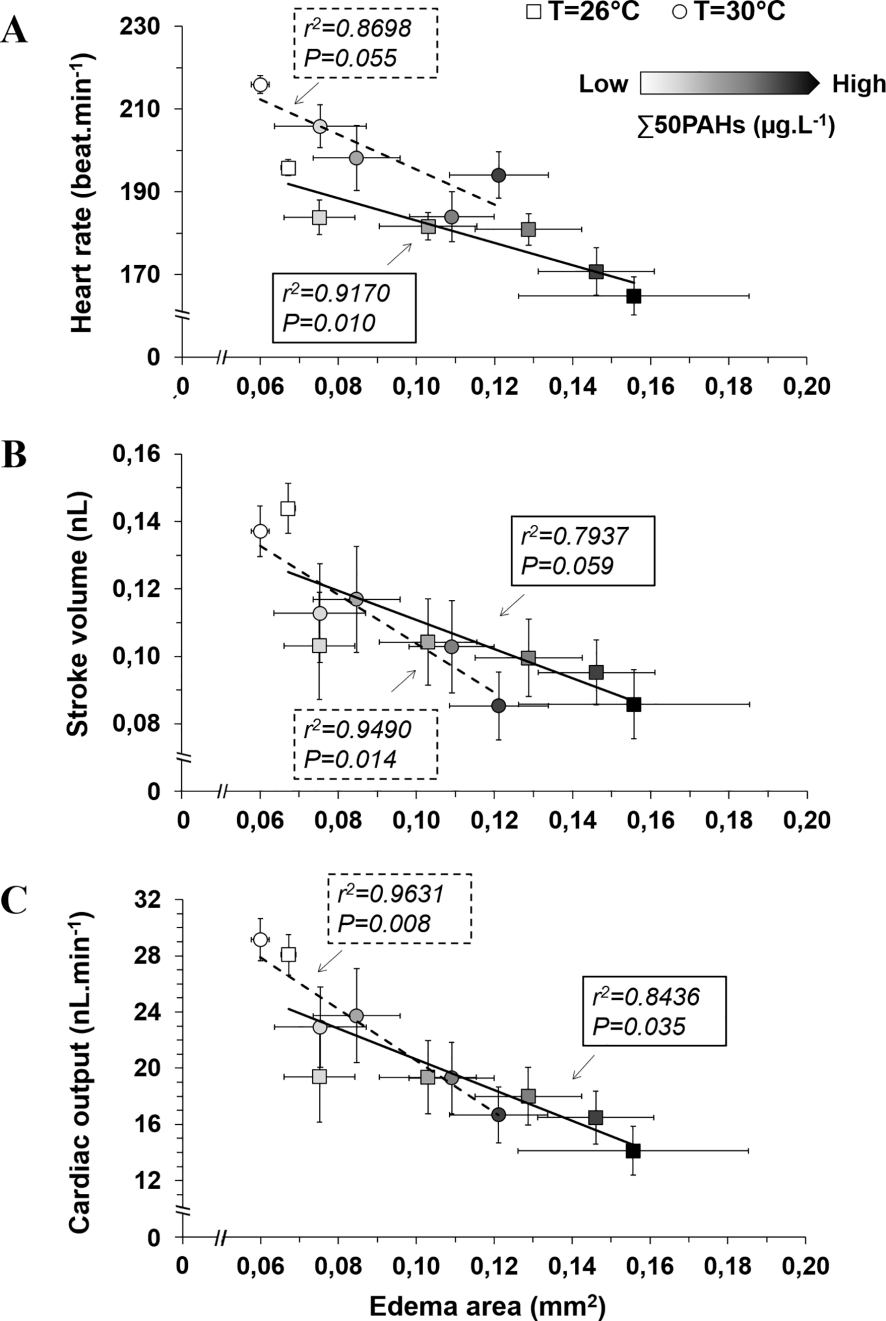
Correlation between cardiac variables and extent of edema in mahi larvae under combined oil and temperature exposures. N = 20–39 at 26°C and N = 20–56 at 30°C. Data are presented as Mean±SEM. Solid and dashed lines represent linear regressions at 26°C and 30°C, respectively.

A linear relationship between increasing edema area and depression of ventricular function occurred as a function of HEWAF concentration exposure at both rearing temperatures (Fig 4). However, a similar linear trend for heart rate was not significant at the elevated temperature of 30°C (r^2^ = 0.87, P = 0.055) compared to 26°C (r^2^ = 0.89, P = 0.010) (Fig 4A). A decrease in stroke volume was correlated with increased edema severity caused by HEWAF exposure in larvae raised at 30°C (r^2^ = 0.95, P = 0.014) (Fig 4B). Reduction of cardiac output by HEWAF exposure was significantly linked to increasing edema area in larvae raised at both rearing temperatures (Fig 4C).

## Discussion

Despite the rich literature of the impact of *DWH* oil on the cardiomorphogenesis of resident Gulf of Mexico fish [5,18,23,27], studies investigating multiple environmental stressors (e.g. combinations of oil exposure and temperatures and their synergies) are still lacking. This current study has measured significant crude oil-induced disruption of cardiac physiology (e.g. depression of cardiac output) in larvae of pelagic mahi, adding to previously documented effects such as pericardial edema and bradycardia. This study study has demonstrated not only that weathered *DWH* oil induced a high incidence of edemas, strong bradycardia and considerable reduction of stroke volume and cardiac output in larvae, but also that the severity of these functional impairments induced by oil exposure was not necessary amplified when combined with elevated rearing temperature during short term exposure.

### Crude oil influence on larval ventricular function

Crude oil derived PAHs are highly toxic to embryonic and larval fish [18,19,25,27,28,30,49,50]. Not surprisingly, then, mahi larvae at even low PAH concentrations displayed the anticipated morphological and functional disorders. These included edema, intrapericardial hematomas, bradycardias and reduced stroke volume and cardiac output at 26°C. Our data also imply a PAH concentration-dependent trend, with greater functional cardiac depression at PAH concentrations higher than 12.4 μg·L^-1^.

Bradycardias and irregular arrhythmia occurred in Pacific herring, *Clupea pallasi*, exposed to Alaska North Slope crude oil, following the 1989 *Exxon Valdez* oil spill [30]. These effects also followed exposure to the 2007 *Cosco Busan* oil spill in San Francisco Bay [50]. Cardiac output was reduced by ~40% in red drum larvae exposed to weathered oil from *DWH* (∑50PAHs = 1.8–2.2 μg·L^-1^) [14]. In zebrafish larvae exposed to Iranian heavy crude oil, ventricular diastolic diameter and contractility decreased by 9% and 43%, respectively, while atrial diameters were unaffected [32]. Abnormal heart alignment or “looping” [5,18] and cardiac muscle excitation or conduction deficiency will likely influence blood ejection fraction, and could account for incomplete ventricular relaxation. These effects, in turn, would lead to a reduction in stroke volume and cardiac output as observed in mahi in the present study or in red drum larvae [14]. Indeed, an increase of atrio-ventricular angle or change in heart shape might impair cardiac pumping efficiency and consequently impair the heart’s capacity to cope with sudden increases in blood transport demand. Such changes might result from the environmental stressors of oxygen and temperature resulting from natural or anthropogenic causes.

Interestingly, although differences exist in terms of physical scale, developmental stage sensitivity and measurement methods, there is a similar impairment of cardiac function in sub-adult mahi exposed to weathered *DWH* oil (∑50PAHs = 9.6 μg·L^-1^) at 26°C [33]. Stroke volume and cardiac output in that study decreased by 44 and 39%, respectively, after a 24-hour oil exposure, while heart rate remained unchanged. Nelson et al. (2016) suggested that a disruption in excitation-contraction coupling in cardiomyocytes might be responsible for the cardiac output depression. In support of this notion, ventricular cardiomyocytes of juvenile bluefin tuna (*Thunnus ortientalis*) and yellowfin tuna (*Thunnus albacares*) displayed decreasing calcium current and cycling, and therefore a depletion in contractile machinery, in response to *DWH* oil [29]. Various genes affecting cellular calcium levels and cardiac muscle contraction are downregulated in larval mahi [23] and larval Atlantic haddock (*Melanogrammus aeglefinus*) [22] after oil exposure, and might be responsible for the depression in stroke volume and cardiac output.

Depression of cardiac output and edema severity were highly correlated, irrespective of rearing temperature. A failure in the functional machinery of the cardiac system can result in edema formation in fish larvae [11,22]. Indeed, functional cardiac phenotypes were mostly detected and coincided with abnormal fluid accumulation in pericardium and/or yolk sac sinus. A modest failure in circulatory function might impair osmoregulation in fishes and consequently induce edema [11,51]. Our data support these predictions. From a physiological point of view, edema formation may in turn lead to elevated pressure in the peritoneal or pericardial cavity, making cardiac filling more difficult–essentially, producting a pericardial edema-induced “cardiac tamponade”. End-diastolic events in particular will be the most impacted. However, additional experimentation is required to reveal the direct functional link between oil-induced edema and cardiac function. Regardless of the mode of actions, oil exposure, which impairs cardiac function, also suppresses maximal oxygen uptake, aerobic scope and swimming performance of mahi [27,52].

### Synergetic influence of temperature and crude oil on larval fish

Cardiac performance in fish embryos and larvae is affected by temperature [43,53–56] and by crude oil related PAHs [5,14,18,19,25,26,30,50,57]. The current and previous studies have demonstrated that crude oil-related PAHs considerably affect physiological performance such as heart contractile function, stroke volume and cardiac output in fish early life stages. However, the synergy and antagonism between temperature and the toxic responses of fish exposed to crude oil have only infrequently been studied [48,58,59]. Hence, the question arises: *“What is the influence of elevated temperature on cardiac performance in fish larvae exposed to crude oil*?*”*

In answering this question, we first must note that elevated temperature favors degradation of PAHs by transformation and volatilization. These compounds are additionally modified following their uptake by fish larvae [60]. In the present study, 9 to 30% and 15 to 32% reduction of PAHs from the initial concentration occurred after 24 hours at 26°C and 30°C, respectively. This degradation might modify larval physiological responses observed at the normal temperature of 26°C compared to 30°C at similar PAH concentrations. Regarding whole body morphology in unexposed fish, elevated temperature typically enhances larval development until an optimal temperature is exceeded [43]. Elevated temperature below this threshold is generally accompanied by bigger size larvae, earlier hatching, faster absorption of vitelline reserve and more rapid advancement through successive larval stages [43,61–66]. In contrast, crude oil related PAHs slowed development and growth, increase edemas and their severity (e.g. pericardium, yolk sac), caused various abnormalities (e.g. craniofacial, spinal), and modified future feeding and swimming behavior. All of these effects are likely to have deleterious consequences on survival [13,14,18,19,21,22,67]. However, no evidence emerged that warmer temperature played a protective role overall morphology of larvae. Indeed, the additive effect of warmer temperature and higher PAHs concentration (> 30.4 μg.L^-1^) leading to the strongest negative impact (mortality) may be due to insufficiency in energy reserves (Pasparakis et al., 2016).

Regarding cardiac physiology, the elevated temperature in 56 hpf larvae increased heart rate by 10%, while stroke volume and cardiac output were not significantly affected in control groups. These results are consistent with previous published observations in mahi [43]. When exposed to 30°C, functional cardiotoxicity (stroke volume and cardiac output) was not significantly amplified in mahi larvae, while morphological variables influencing the heart function were slightly disrupted.

The impact of oil is sufficiently high at concentrations over 12.4 μg·L^-1^ such that the temperature effects may be minor in the regulation of cardiac function compared to the lethal effects of oil. As well, it is difficult to fully understand the true impact of elevated temperature based on only short term exposure (56 hours). Indeed, it is difficult to draw clear conclusions from MLR models (low r squared, low P value), both because of the biological variability that is increased with concentrations parameters and the small number of regressors (i.e. temperature). Furthermore, even if elevated temperature might slightly enhance development time of larvae [43], it does not seem to have a very large influence on the cardiotoxicity of weathered oil in mahi larvae, especially during the very short term exposures used in this study. PAH concentrations were more of an influencing factor than temperature.

Functional phenotypes involved in cardiac regulation are consistent with transcriptomic responses observed in larval mahi exposed to the same crude oil at the optimal temperature of 26°C [23]. As previously described, strong upregulation of genes not associated with the AhR pathway were observed, but it has also been found that the detoxification mechanisms were significantly upregulated in exposed larvae [23,68]. Strong induction of AhR detoxifying systems are not only energetically costly for organisms but also might be responsible for cardiac failure in larvae after oil exposure [30,69–71]. Environmental temperature also influences the level of various xenobiotic metabolizing enzyme activities in fish exposed to crude oil [58,59]. Indeed, regulation and induction of these detoxification mechanisms (*cyp1a*, EROD) and several chaperone genes were much higher at elevated temperatures in Atlantic cod, *Gadus morhua* [59] and in polar cod, *Boreogadus saida* [58]. These enzymatic modulations might cause the slight (but insignificant) increase or comparable functional change in cardiac modulation at elevated temperature compared to 26°C in larval mahi.

Overall, our findings indicate that the interaction between elevated temperature and crude oil exposure acted differentially on the cardiac system of larval fish. PAH concentrations of crude oil appear to play a greater role on the metabolic demand and regulation of ventricular function in mahi larvae than the temperature factor during these short term exposures.

### Directions for future studies

Additional research is needed to further elucidate the complex interactions between rearing temperature, PAH toxicants, and cardiac form and function. Future investigations should be undertaken with more developed larvae to better understand the mechanisms underlying these cardiac adjustments, in particular at developmental stages where the relationship between both cardiac and circulatory systems is established and oxygen demand becomes more important for organisms. Furthermore, a sudden, acute change of temperature would be more representative of environmental conditions.

Moreover, crude oil is a complex mixture of thousands of compounds generally characterized by its aromatic hydrocarbons fraction (i.e. BTEX, PAH). While this aromatic fraction is assumed to be a primary determinant of oil toxicity [72,73], other aromatic compounds from the unresolved complex mixture (UCM) may also strongly contribute to this toxicity by eliciting nonspecific narcotic toxic responses [74,75]. Most studies, including our present work, have characterized the oil fraction by measuring PAH as the basic metric for comparison of toxicity testing, but additive effects of the mixture should be considered in future studies.

## Conclusion

A combination of elevated temperature and crude oil exposure has a differential effect on severity of morphological impairments and cardiac performance in mahi. Elevated temperature influenced solubility, bioavailability of some compounds and gas transport. However, elevated water temperature did not protect the heart from functional oil-induced modifications, such as bradycardia. Stroke volume was unaffected, and cardiac output only slightly affected, by elevated temperature. Furthermore, reduction of edema in the pericardium and yolk sinus cavity occurred at 30°C. Nevertheless, a potential protective role of warmer temperature cannot be supported, because of a lethal effect observed at higher exposure concentrations. Quite the contrary, this smaller area of edema is probably morphologically related to the advanced development in time of larvae, in which yolk sac absorption becomes more important and nutritional and energetic reserves decrease, leading to increasing vulnerability of larval fish. We suggest that elevated temperature may decrease larval resilience to oil exposure by amplifying energetic costs to organisms. However, it is difficult to demonstrate this effect of increased energetic costs with short term exposure. This study adds growing evidence to the importance of considering environmental factors in risk assessment of anthropogenic pollutants on physiology and fitness of aquatic organisms. In the current context of global warming, environmental water temperature should be considered as a key variable and thermally related physiological challenges should be used in toxicity testing to further explore physiological and ecological impacts of these interactions.

## Supporting information

S1 Fig Micrograph of larval mahi with a representative control and oil-exposed larvae at both rearing temperature, 26°C and 30°C. (TIF)

S1 Table Water quality parameters during experiments at initial (8 hpf), final (32 hpf) exposure time and after transfer in clean sea water. Values represent Mean±SEM.
(DOCX)

S2 Table Number of total individuals used for each morphological and physiological measurement. (DOCX)

S3 Table Chemical characterization of 50 PAHs found in HEWAF solutions used during fish embryonic exposure at both rearing temperatures 26°C and 30°C. (DOCX)
