# Supplementary figures and images for: Combined effects of elevated temperature and Deepwater Horizon oil exposure on the cardiac performance of larval mahi-mahi, Coryphaena hippurus
Source: PLoS One. 2018 Oct 17;13(10):e0203949. doi: 10.1371/journal.pone.0203949 (PMC6192557; doi:10.1371/journal.pone.0203949)

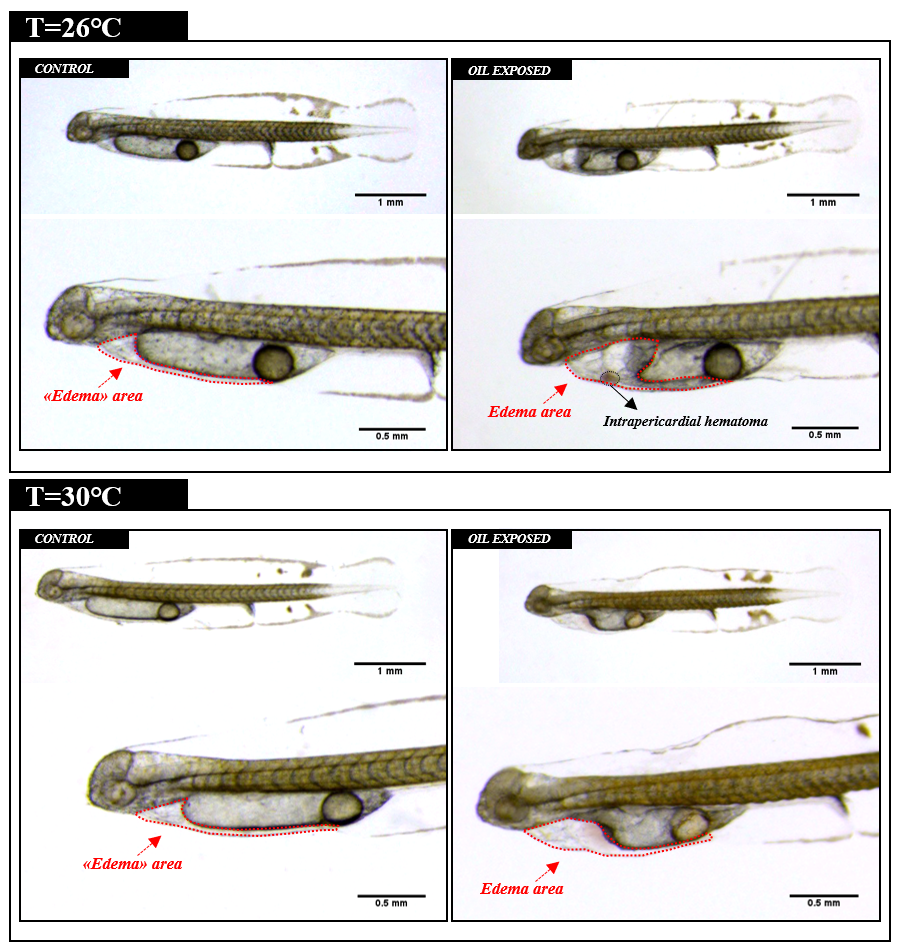

Supplement: S1 Fig — (TIF) [file pone.0203949.s001.tif]
