# Supplementary material for: Combined effects of elevated temperature and Deepwater Horizon oil exposure on the cardiac performance of larval mahi-mahi, Coryphaena hippurus
Source: PLoS One. 2018 Oct 17;13(10):e0203949. doi: 10.1371/journal.pone.0203949 (PMC6192557; doi:10.1371/journal.pone.0203949)
Supplement: S1 Table — Values represent Mean±SEM. (DOCX) [file pone.0203949.s002.docx]

|  |  | Temperature (℃) | | | DO (mg.L^-1^) | | | | pH (s.u.) | | | Salinity (ppt) | | |
| --- | --- | --- | --- | --- | --- | --- | --- | --- | --- | --- | --- | --- | --- | --- |
|  | Geometric means ∑PAHs (µg.L^-1^) | Initial | Final | Transfer (clean seawater) | Initial | Final | Transfer (clean seawater) | Initial | | Final | Transfer (clean seawater) | Initial | Final | Transfer (clean seawater) |
| Normal temperature  26℃ | 0.0 | 25.90 ± 0.19 | 25.92 ± 0.21 | 26.05 ± 0.25 | 6.72 ± 0.06 | 6.50 ± 0.11 | 6.73 ± 0.01 | 8.10 ± 0.05 | | 8.11 ± 0.05 | 8.11 ± 0.03 | 34.20 ± 0.37 | 34.20 ± 0.37 | 34.5 ± 0.50 |
|  | 3.0 | 26.40 ± 0.00 | 26.40 ± 0.00 |  | 6.82 ± 0.06 | 6.70 ± 0.06 |  | 7.92 ± 0.00 | | 7.92 ± 0.00 |  | 33.00 ± 0.00 | 33.00 ± 0.00 |  |
|  | 7.4 | 26.40 ± 0.00 | 26.40 ± 0.00 |  | 6.80 ± 0.05 | 6.70 ± 0.05 |  | 7.92 ± 0.01 | | 7.94 ± 0.00 |  | 33.00 ± 0.00 | 33.00 ± 0.00 |  |
|  | 12.4 | 26.10 ± 0.20 | 25.70 ± 0.20 |  | 6.63 ± 0.16 | 6.32 ± 0.16 |  | 8.13 ± 0.00 | | 8.14 ± 0.00 |  | 34.00 ± 0.50 | 34.00 ± 0.50 |  |
|  | 31.2 | 26.07 ± 0.28 | 26.03 ± 0.37 |  | 6.78 ± 0.04 | 6.62 ± 0.08 |  | 8.07 ± 0.08 | | 8.08 ± 0.07 |  | 34.33 ± 0.67 | 34.33 ± 0.67 |  |
|  | 44.1 | 25.70 ± 0.05 | 25.60 ± 0.05 |  | 6.57 ± 0.20 | 6.38 ± 0.20 |  | 8.13 ± 0.02 | | 8.09 ± 0.02 |  | 34.00 ± 0.00 | 34.00 ± 0.00 |  |
|  |  |  |  |  |  |  |  |  | |  |  |  |  |  |
| Elevated temperature  30℃ | 0.0 | 29.28 ± 0.03 | 29.51 ± 0.03 | 29.6 ± 0.05 | 6.67 ± 0.02 | 6.23 ± 0.02 | 6.72 ± 0.02 | 8.09 ± 0.01 | | 8.08 ± 0.01 | 7.89 ± 0.29 | 33.23 ± 0.08 | 33.55 ± 0.10 | 33.5 ± 0.50 |
|  | 3.1 | 29.50 ± 0.10 | 29.30 ± 0.10 |  | 6.72 ± 0.12 | 6.49 ± 0.12 |  | 7.70 ± 0.04 | | 7.78 ± 0.04 |  | 34.00 ± 0.00 | 34.00 ± 0.00 |  |
|  | 7.8 | 29.50 ± 0.10 | 29.30 ± 0.10 |  | 6.72 ± 0.12 | 6.49 ± 0.12 |  | 7.66 ± 0.06 | | 7.78 ± 0.06 |  | 34.00 ± 0.00 | 34.00 ± 0.00 |  |
|  | 14.9 | 29.41 ± 0.03 | 29.62 ± 0.01 |  | 6.41 ± 0.02 | 6.04 ± 0.01 |  | 8.08 ± 0.00 | | 8.10 ± 0.00 |  | 33.00 ± 0.00 | 33.00 ± 0.00 |  |
|  | 26.6 | 29.12 ± 0.02 | 29.53 ± 0.03 |  | 6.67 ± 0.00 | 6.15 ± 0.01 |  | 8.15 ± 0.00 | | 8.08 ± 0.00 |  | 33.20 ± 0.11 | 33.87 ± 0.09 |  |

**S1 Table**. **Water quality parameters during experiments at initial (8 hpf), final (32 hpf) exposure time and after transfer in clean sea water.** Values represent Mean±SEM.
