# Supplementary material for: Combined effects of elevated temperature and Deepwater Horizon oil exposure on the cardiac performance of larval mahi-mahi, Coryphaena hippurus
Source: PLoS One. 2018 Oct 17;13(10):e0203949. doi: 10.1371/journal.pone.0203949 (PMC6192557; doi:10.1371/journal.pone.0203949)
Supplement: S2 Table — (DOCX) [file pone.0203949.s003.docx]

**S2 Table. Number of total individuals used for each morphological and physiological measurement.**

|  | Geometric means ∑PAHs (µg.L^-1^) | Exposure replicate | Number larvae used for measurement* |
| --- | --- | --- | --- |
| Normal temperature 26℃ | 0 | 4 | 39 |
|  | 3 | 3 | 20 |
|  | 7.40 | 3 | 24 |
|  | 12.4 | 3 | 16 |
|  | 31.2 | 3 | 20 |
|  | 44.1 | 3 | 18 |
| Elevated temperature 30℃ | 0 | 3 | 56 |
|  | 3.1 | 3 | 18 |
|  | 7.8 | 3 | 20 |
|  | 14.9 | 3 | 22 |
|  | 26.6 | 3 | 25 |
| * Larvae were sampled per replicates and pooled for measurement due to low quality videos (See Materials and Methods). | | | |
