# Supplementary material for: Combined effects of elevated temperature and Deepwater Horizon oil exposure on the cardiac performance of larval mahi-mahi, Coryphaena hippurus
Source: PLoS One. 2018 Oct 17;13(10):e0203949. doi: 10.1371/journal.pone.0203949 (PMC6192557; doi:10.1371/journal.pone.0203949)
Supplement: S3 Table — (DOCX) [file pone.0203949.s004.docx]

**S3 Table. Chemical characterization of 50 PAHs found in HEWAF solutions used during fish embryonic exposure at both rearing temperatures 26℃ and 30℃.**

|  | **Temperature trials** | | **Ambient "26℃"** | | | | | | | | | | | | **High "30℃"** | | | | | | | | | | | |
| --- | --- | --- | --- | --- | --- | --- | --- | --- | --- | --- | --- | --- | --- | --- | --- | --- | --- | --- | --- | --- | --- | --- | --- | --- | --- | --- |
|  | **Concentrations (µg.L^-1^)** | **Detection Limit (µg.L^-1^)** | **Initial** | | | | | | **Final** | | | | | | **Initial** | | | | | | **Final** | | | | | |
| **PAH structure** | **Compounds** |  | Control | 0.5% | 1 % | 2 % | 4 % | 8 % | Control | 0.5% | 1 % | 2 % | 4 % | 8 % | Control | 0.5% | 1 % | 2 % | 4 % | 8 % | Control | 0.5% | 1 % | 2 % | 4 % | 8 % |
| 2-ring | Naphthalene | 0.012 | 0.00 | ND | 0.02 | 0.01 | 0.01 | ND | 0.01 | ND | 0.01 | 0.01 | 0.02 | ND | 0.00 | 0.01 | ND | 0.01 | 0.01 | ND | ND | ND | ND | ND | 0.01 | ND |
|  | C1-Naphthalenes | 0.012 | ND | ND | 0.03 | 0.01 | 0.02 | ND | ND | ND | 0.02 | ND | 0.03 | ND | ND | ND | ND | 0.01 | 0.01 | 0.01 | ND | ND | ND | ND | 0.01 | ND |
|  | C2-Naphthalenes | 0.012 | ND | ND | 0.06 | 0.05 | 0.13 | 0.14 | ND | ND | 0.06 | 0.04 | 0.10 | 0.08 | ND | ND | 0.03 | 0.05 | 0.09 | 0.17 | ND | ND | ND | 0.04 | 0.07 | 0.09 |
|  | C3-Naphthalenes | 0.012 | ND | 0.06 | 0.14 | 0.18 | 0.48 | 0.66 | ND | 0.04 | 0.12 | 0.14 | 0.37 | 0.44 | ND | 0.07 | 0.13 | 0.23 | 0.43 | 0.73 | ND | 0.04 | 0.09 | 0.15 | 0.29 | 0.44 |
|  | C4-Naphthalenes | 0.012 | ND | 0.09 | 0.26 | 0.29 | 0.92 | 1.07 | ND | ND | 0.19 | 0.22 | 0.69 | 0.88 | ND | 0.11 | 0.22 | 0.40 | 0.78 | 1.37 | ND | ND | 0.15 | 0.28 | 0.54 | 0.84 |
|  | Biphenyl | 0.010 | 0.01 | 0.02 | 0.03 | 0.02 | 0.03 | 0.03 | 0.01 | 0.02 | 0.03 | 0.02 | 0.03 | 0.02 | 0.01 | 0.02 | 0.01 | 0.02 | 0.02 | 0.03 | 0.01 | 0.02 | 0.02 | 0.02 | 0.02 | 0.02 |
| 3-ring | Dibenzofuran | 0.018 | ND | ND | ND | 0.01 | 0.01 | ND | ND | ND | ND | ND | 0.01 | ND | ND | ND | ND | 0.01 | 0.01 | 0.02 | ND | ND | ND | ND | 0.01 | 0.01 |
|  | Acenaphthylene | 0.014 | ND | ND | ND | ND | ND | ND | ND | ND | ND | ND | ND | ND | ND | ND | ND | ND | ND | ND | ND | ND | ND | ND | ND | ND |
|  | Acenaphthene | 0.018 | ND | ND | ND | 0.01 | ND | ND | ND | ND | ND | ND | ND | ND | ND | ND | ND | 0.01 | ND | 0.01 | ND | ND | ND | ND | 0.00 | ND |
|  | Fluorene | 0.015 | ND | 0.01 | 0.02 | 0.03 | 0.06 | 0.09 | ND | 0.01 | 0.02 | 0.02 | 0.05 | 0.06 | ND | 0.01 | 0.02 | 0.04 | 0.06 | 0.10 | 0.00 | 0.01 | 0.02 | 0.03 | 0.05 | 0.07 |
|  | C1-Fluorenes | 0.015 | ND | 0.06 | 0.13 | 0.25 | 0.42 | 0.60 | ND | 0.05 | 0.11 | 0.18 | 0.36 | 0.43 | ND | 0.07 | 0.11 | 0.25 | 0.41 | 0.79 | ND | 0.06 | 0.10 | 0.18 | 0.31 | 0.51 |
|  | C2-Fluorenes | 0.015 | ND | 0.19 | 0.44 | 0.55 | 1.51 | 1.87 | ND | 0.15 | 0.38 | 0.57 | 1.30 | 1.39 | ND | 0.21 | 0.40 | 0.74 | 1.22 | 2.41 | ND | 0.20 | 0.36 | 0.57 | 0.94 | 1.67 |
|  | C3-Fluorenes | 0.015 | ND | 0.17 | 0.63 | 1.07 | 1.98 | 2.63 | ND | 0.18 | 0.59 | 0.80 | 1.87 | 2.15 | ND | ND | 0.56 | 1.07 | 1.86 | 3.51 | ND | ND | 0.54 | 0.80 | 1.39 | 2.37 |
|  | Anthracene | 0.014 | ND | ND | ND | ND | 0.02 | ND | ND | ND | 0.01 | ND | 0.02 | ND | ND | ND | 0.01 | ND | 0.02 | 0.04 | ND | ND | ND | ND | 0.03 | ND |
|  | Phenanthrene | 0.020 | 0.00 | 0.07 | 0.13 | 0.24 | 0.50 | 0.91 | ND | 0.06 | 0.12 | 0.17 | 0.45 | 0.65 | 0.00 | 0.08 | 0.15 | 0.31 | 0.51 | 0.96 | 0.01 | 0.07 | 0.13 | 0.24 | 0.41 | 0.70 |
|  | C1-Phenanthrenes/Anthracenes | 0.020 | ND | 0.34 | 0.73 | 1.20 | 2.83 | 4.72 | ND | 0.30 | 0.61 | 0.88 | 2.43 | 3.71 | ND | 0.38 | 0.75 | 1.59 | 2.78 | 5.05 | ND | 0.31 | 0.62 | 1.24 | 2.11 | 3.70 |
|  | C2-Phenanthrenes/Anthracenes | 0.020 | ND | 0.59 | 1.30 | 2.15 | 4.55 | 7.69 | ND | 0.48 | 1.15 | 1.67 | 4.27 | 6.14 | ND | 0.59 | 1.45 | 2.84 | 4.43 | 7.97 | ND | 0.54 | 1.26 | 2.17 | 3.34 | 5.81 |
|  | C3-Phenanthrenes/Anthracenes | 0.020 | ND | 0.37 | 0.76 | 1.41 | 3.00 | 5.41 | ND | 0.33 | 0.67 | 1.04 | 2.96 | 4.50 | ND | 0.40 | 0.82 | 1.79 | 2.80 | 5.38 | ND | 0.37 | 0.73 | 1.26 | 2.18 | 3.88 |
|  | C4-Phenanthrenes/Anthracenes | 0.020 | ND | 0.19 | 0.61 | 0.89 | 2.24 | 3.19 | ND | 0.23 | 0.52 | 0.64 | 1.92 | 2.74 | ND | 0.25 | 0.65 | 1.18 | 2.02 | 3.57 | ND | 0.24 | 0.47 | 0.75 | 1.41 | 2.73 |
|  | Dibenzothiophene | 0.015 | ND | 0.01 | 0.02 | 0.03 | 0.07 | 0.13 | ND | 0.01 | 0.02 | 0.02 | 0.06 | 0.09 | ND | 0.01 | 0.02 | 0.04 | 0.07 | 0.14 | ND | 0.01 | 0.02 | 0.03 | 0.05 | 0.09 |
|  | C1-Dibenzothiophenes | 0.015 | ND | 0.07 | 0.15 | 0.25 | 0.59 | 1.00 | ND | 0.06 | 0.13 | 0.17 | 0.50 | 0.71 | ND | 0.08 | 0.15 | 0.33 | 0.52 | 0.97 | ND | 0.06 | 0.12 | 0.23 | 0.42 | 0.75 |
|  | C2-Dibenzothiophenes | 0.015 | ND | 0.17 | 0.38 | 0.59 | 1.52 | 2.28 | ND | 0.14 | 0.34 | 0.43 | 1.37 | 1.84 | ND | 0.18 | 0.38 | 0.80 | 1.38 | 2.56 | ND | 0.16 | 0.32 | 0.60 | 1.04 | 1.87 |
|  | C3-Dibenzothiophenes | 0.015 | ND | 0.17 | 0.36 | 0.54 | 1.33 | 1.97 | ND | 0.13 | 0.29 | 0.42 | 1.27 | 1.72 | ND | 0.16 | 0.37 | 0.73 | 1.33 | 2.68 | ND | 0.16 | 0.32 | 0.52 | 0.96 | 1.74 |
|  | C4-Dibenzothiophenes | 0.015 | ND | 0.17 | 0.11 | 1.10 | 1.87 | 1.94 | ND | ND | 0.12 | 0.16 | 1.75 | 1.71 | ND | 0.06 | 0.15 | 1.10 | 1.47 | 2.26 | ND | ND | 0.12 | 0.16 | 1.23 | 1.70 |
| 4-ring | Benzo(b)fluorene | 0.004 | ND | ND | 0.01 | 0.02 | 0.04 | 0.06 | ND | ND | 0.01 | 0.02 | 0.03 | 0.05 | ND | 0.01 | 0.01 | 0.02 | 0.04 | 0.07 | ND | ND | 0.01 | 0.02 | 0.03 | 0.06 |
|  | Fluoranthene | 0.018 | ND | ND | 0.01 | 0.02 | 0.03 | 0.04 | ND | ND | 0.01 | 0.02 | 0.03 | 0.03 | ND | 0.01 | 0.01 | 0.02 | 0.03 | 0.05 | ND | 0.01 | 0.01 | 0.02 | 0.02 | 0.04 |
|  | Pyrene | 0.014 | ND | 0.01 | 0.02 | 0.03 | 0.08 | 0.12 | ND | 0.02 | 0.02 | 0.04 | 0.07 | 0.12 | ND | 0.01 | 0.02 | 0.05 | 0.08 | 0.14 | ND | 0.01 | 0.02 | 0.04 | 0.06 | 0.11 |
|  | C1-Fluoranthenes/Pyrenes | 0.018 | ND | 0.05 | 0.11 | 0.17 | 0.41 | 0.57 | ND | 0.04 | 0.10 | 0.12 | 0.39 | 0.51 | ND | 0.05 | 0.12 | 0.23 | 0.36 | 0.71 | ND | 0.06 | 0.10 | 0.16 | 0.26 | 0.53 |
|  | C2-Fluoranthenes/Pyrenes | 0.018 | ND | ND | 0.18 | 0.33 | 0.61 | 0.94 | ND | 0.06 | 0.15 | 0.25 | 0.59 | 0.71 | ND | 0.07 | 0.15 | 0.33 | 0.59 | 1.12 | ND | ND | 0.14 | 0.25 | 0.42 | 0.77 |
|  | C3-Fluoranthenes/Pyrenes | 0.018 | ND | ND | 0.20 | 0.41 | 0.86 | 1.05 | ND | ND | ND | 0.30 | 0.81 | 0.98 | ND | ND | 0.20 | 0.41 | 0.76 | 1.47 | ND | ND | 0.19 | 0.30 | 0.52 | 1.04 |
|  | C4-Fluoranthenes/Pyrenes | 0.018 | ND | ND | 0.21 | 0.45 | 0.85 | 1.20 | ND | ND | ND | 0.29 | 0.76 | 1.09 | ND | ND | 0.15 | 0.45 | 0.76 | 1.49 | ND | ND | 0.22 | 0.29 | 0.56 | 1.07 |
|  | Naphthobenzothiophene | 0.008 | ND | 0.02 | 0.03 | 0.07 | 0.15 | 0.27 | ND | 0.01 | 0.03 | 0.05 | 0.13 | 0.22 | ND | 0.02 | 0.04 | 0.09 | 0.16 | 0.30 | ND | 0.02 | 0.03 | 0.07 | 0.12 | 0.22 |
|  | C1-Naphthobenzothiophenes | 0.008 | ND | ND | 0.14 | 0.27 | 0.58 | 1.09 | ND | 0.07 | 0.14 | 0.20 | 0.49 | 0.91 | ND | 0.07 | 0.17 | 0.35 | 0.58 | 1.17 | ND | 0.08 | 0.15 | 0.26 | 0.45 | 0.84 |
|  | C2-Naphthobenzothiophenes | 0.008 | ND | ND | 0.16 | 0.41 | 0.72 | 1.29 | ND | 0.09 | 0.16 | 0.31 | 0.68 | 0.98 | ND | 0.09 | 0.21 | 0.41 | 0.74 | 1.37 | ND | ND | 0.21 | 0.31 | 0.54 | 1.01 |
|  | C3-Naphthobenzothiophenes | 0.008 | ND | ND | ND | 0.23 | 0.49 | 0.92 | ND | ND | ND | ND | 0.51 | ND | ND | ND | ND | 0.23 | 0.49 | 0.92 | ND | ND | ND | ND | 0.34 | 0.59 |
|  | C4-Naphthobenzothiophenes | 0.008 | ND | ND | ND | ND | 0.39 | ND | ND | ND | ND | ND | 0.37 | ND | ND | ND | ND | ND | 0.35 | 0.58 | ND | ND | ND | ND | 0.26 | ND |
|  | Benz(a)anthracene | 0.010 | 0.01 | 0.01 | ND | ND | 0.02 | ND | 0.01 | 0.01 | 0.01 | ND | ND | ND | 0.01 | 0.01 | ND | ND | ND | ND | ND | 0.01 | ND | ND | ND | 0.02 |
|  | Chrysene | 0.014 | ND | 0.07 | 0.14 | 0.24 | 0.50 | 0.90 | ND | 0.06 | 0.11 | 0.19 | 0.47 | 0.71 | ND | 0.07 | 0.16 | 0.33 | 0.56 | 1.01 | ND | 0.06 | 0.14 | 0.25 | 0.42 | 0.75 |
|  | C1-Chrysenes | 0.014 | ND | 0.14 | 0.25 | 0.45 | 1.00 | 1.71 | ND | 0.12 | 0.22 | 0.34 | 0.92 | 1.47 | ND | 0.13 | 0.27 | 0.60 | 1.10 | 1.91 | ND | 0.13 | 0.25 | 0.41 | 0.80 | 1.39 |
|  | C2-Chrysenes | 0.014 | ND | 0.11 | 0.25 | 0.44 | 1.00 | 1.57 | ND | 0.12 | 0.23 | 0.37 | 0.93 | 1.36 | ND | 0.13 | 0.27 | 0.56 | 0.99 | 1.84 | ND | 0.15 | 0.28 | 0.43 | 0.73 | 1.38 |
|  | C3-Chrysenes | 0.014 | ND | ND | ND | 0.33 | 0.63 | 0.97 | ND | ND | ND | 0.23 | 0.56 | 0.70 | ND | ND | 0.21 | 0.33 | 0.59 | 1.15 | ND | ND | ND | 0.23 | 0.45 | 0.75 |
|  | C4-Chrysenes | 0.014 | ND | ND | ND | ND | ND | ND | ND | ND | ND | ND | ND | ND | ND | ND | ND | ND | 0.42 | 0.78 | ND | ND | ND | ND | ND | 0.53 |
| 5+-ring | Benzo(b)fluoranthene | 0.009 | ND | ND | 0.01 | 0.03 | 0.06 | 0.09 | ND | 0.01 | 0.02 | 0.02 | 0.06 | 0.07 | ND | 0.01 | 0.02 | 0.03 | 0.06 | 0.14 | ND | ND | 0.02 | 0.02 | 0.04 | 0.09 |
|  | Benzo(k)fluoranthene | 0.010 | ND | ND | ND | ND | ND | ND | ND | ND | ND | ND | ND | ND | ND | ND | ND | ND | ND | ND | ND | ND | ND | ND | ND | ND |
|  | Benzo(a)fluoranthene | 0.004 | ND | ND | ND | ND | ND | ND | ND | ND | ND | ND | ND | ND | ND | ND | ND | ND | ND | ND | ND | ND | ND | ND | ND | ND |
|  | Benzo(e)pyrene | 0.016 | ND | ND | 0.02 | 0.03 | 0.09 | 0.18 | ND | 0.01 | 0.02 | 0.04 | 0.08 | 0.16 | ND | 0.01 | 0.02 | 0.05 | 0.10 | 0.19 | ND | ND | 0.02 | 0.04 | 0.07 | 0.13 |
|  | Benzo(a)pyrene | 0.017 | ND | ND | ND | ND | ND | ND | ND | ND | ND | ND | ND | ND | ND | ND | ND | ND | ND | ND | ND | ND | ND | ND | ND | ND |
|  | Indeno(1,2,3-cd)pyrene | 0.010 | ND | ND | ND | ND | ND | ND | ND | ND | ND | ND | ND | ND | ND | ND | ND | ND | ND | ND | ND | ND | ND | ND | ND | ND |
|  | Dibenz(a,h)anthracene | 0.010 | ND | ND | ND | ND | 0.01 | ND | ND | ND | ND | ND | ND | ND | ND | ND | ND | ND | ND | 0.02 | ND | ND | ND | ND | ND | ND |
|  | Benzo(g,h,i)perylene | 0.012 | ND | ND | ND | 0.01 | 0.01 | 0.03 | ND | ND | ND | ND | 0.01 | ND | ND | ND | ND | 0.01 | 0.01 | 0.03 | ND | ND | ND | ND | 0.01 | 0.02 |
|  |  | **SUM 50PAHs** | **0.02** | **3.15** | **8.01** | **14.80** | **32.63** | **49.33** | **0.03** | **2.80** | **6.73** | **10.34** | **29.73** | **39.33** | **0.02** | **3.36** | **8.39** | **18.02** | **31.00** | **57.19** | **0.02** | **2.77** | **7.15** | **12.31** | **22.89** | **40.32** |
|  | ND: < detection limit | | | | | | | | | | | | | | | | | | | | | | | | |  |
